# Placenta Percreta Resulting in Incomplete Spontaneous Abortion in First Trimester

**Published:** 2014-11-01

**Authors:** Mine Genc, Berhan Genc, Aynur Solak, Oya Nermin Sivrikoz

**Affiliations:** 1 Department of Obstetrics and Gynecology, Sifa University School of Medicine, Izmir, Turkey; 2 Department of Radiology, Sifa University School of Medicine, Izmir, Turkey; 3 Department of Patology, Sifa University School of Medicine, Izmir, Turkey

**Keywords:** Incomplete Abortion, Magnetic Resonance Imaging, Placenta Percreta, Ul-trasonography

## Abstract

Placenta percreta is a rare complication potentially fatal to fetus and the mother. We present here a 41-year-old female patient who underwent curettage for incomplete abortion at 6^th^ week of pregnancy. She had persistent vaginal bleeding for 2 months after the curettage, for which she was treated with hysterectomy. Preoperative ultrasonography and magnetic resonance imaging (MRI) made the diagnosis of placenta percreta. Postoperative pathological examination confirmed this diagnosis.

## Introduction

Placenta accreta (abnormal placentation) is characterized by a regional or insufficient diffusion of decidua basalis. It has three types: placenta accreta where the villi are superficially attached to, but do not invade the uterus; placenta increta where villi invade myometrium; and placenta percreta where placenta crosses full thickness of myometrium and reaches the serosa (1, 2).

Placenta percreta is the most severe form as it invades the serosal layer of the uterus and has a potential to invade adjacent pelvic organs. Its incidence has been increasing with each passing day as a result of an increase in the number of surgical deliveries (3). Hysterectomy is usually needed to control life-threatening bleeding. In selected hemodynamically stable patients, a conservative approach may be tried both to preserve the fertility and to reduce morbidity and the amount of blood transfusion (4).

We report a patient with placenta percreta who presented with continued vaginal bleeding following curettage for incomplete abortion at 6 weeks of pregnancy.

## Case Report

**Fig 1:**
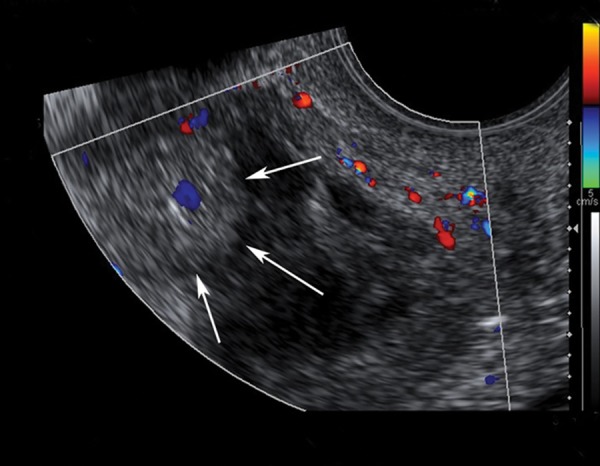
Transvaginal color Doppler interrogation demonstrates a solid mass lesion with smooth contour and a central vascular flow at the anterior wall of the uterine isthmus.

**Fig 2:**
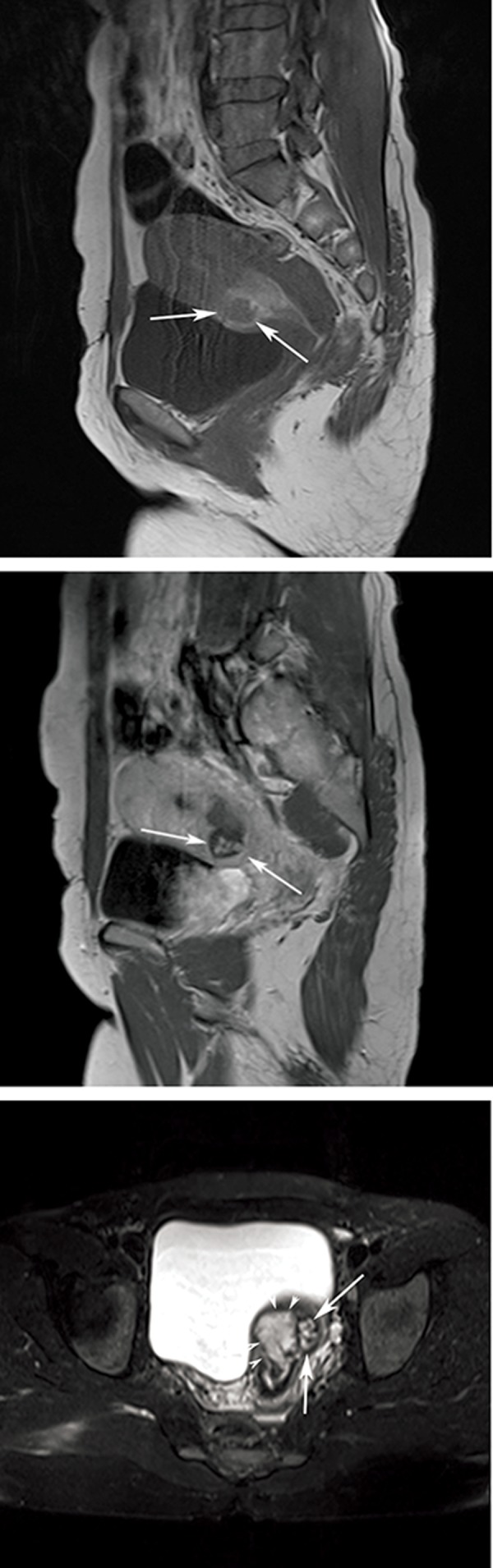
T1W sagittal precontrast image (A) shows residual placental (white arrows) and a hyperintense area consistent with subacute hemorrhage around it at the posterior segment of uterus. T1W sagittal postcontrast image (B) shows contrast uptake in myometrium and placental residue (white arrows). Axial fat suppression T2W image (C) shows placental residue (white arrows) and hemorrhage (arrow heads).

**Fig 3:**
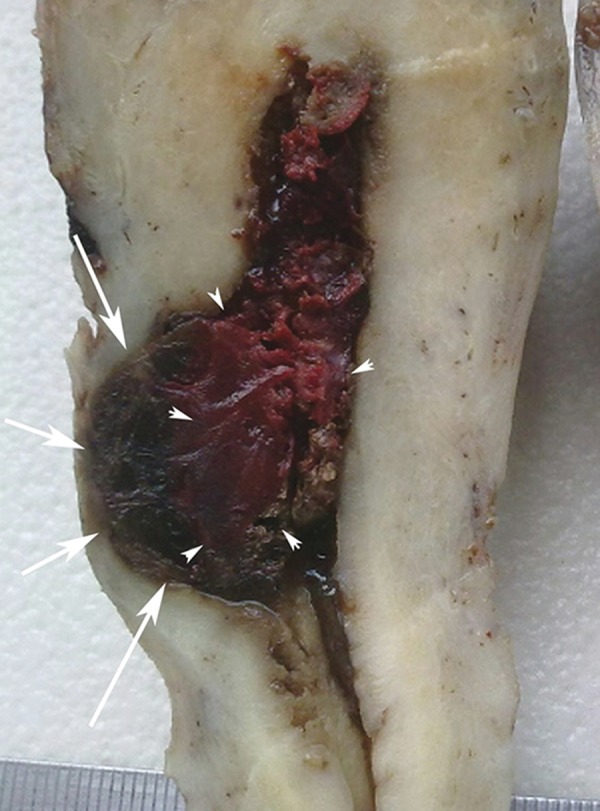
Postoperative hysterectomy material reveals placenta percreta extending to serosa (white arrows) at the level of uterine isthmus and a hematoma opening to endometrial cavity medial to it (arrow heads).

**Fig 4:**
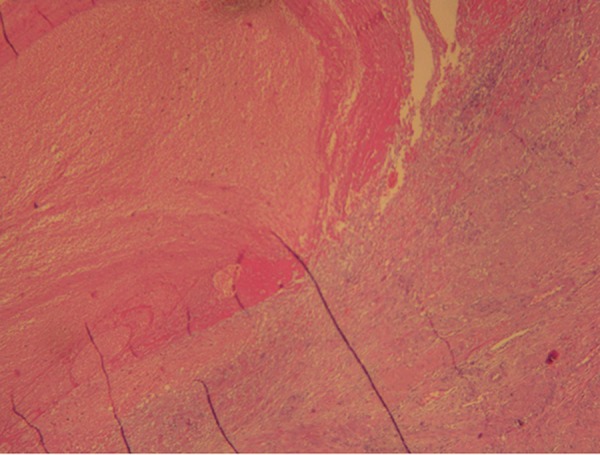
Macrophotography demonstrates hemorrhagic placental residue containing necrotic villi interspersed in muscle tissue.

41-year-old female G3 P2 L1 A1 with history of 2 previous cesarean deliveries, presented to our clinic with protracted vaginal bleeding. It was learnt that she had undergone curettage for incomplete abortion at an outside center 2 months ago, following which she had persistent vaginal bleeding. She did not apply to any healthcare facility because her sociocultural level was low and she resided in a rural area of İzmir, Turkey. She could not have any sexual intercourse as a result of protracted bleeding. Her hemoglobin was 7.1 g/dl, and beta-human chorionic gonadotropin (β-HCG) was 130 mIU/ml. Transabdominal and transvaginal ultrasonography (USG) was used to rule out abortion imminens and extrauterine pregnancy. Transvaginal color Doppler ultrasonography revealed a 20 mm solid mass lesion with smooth contour compressing endometrium anterior to uterine isthmus as well as a dense fluid collection within the cavity (Fig 1). T2-wieghted (T2W) magnetic resonance imaging (MRI) showed hyperintense lesions extending to endometrial cavity at the anterior part of isthmus (Fig 2A). Fat suppression axial T2W images demonstrated fluid collection in the cavity and a hyperintense lesion in the myometrium (Fig 2B). Pre and post contrast T1W sagittal images showed a myometrial mass lesion with localized contrast uptake and a hematoma compressing the cavity (Fig 2C). Considering the elevated β-HCG level, it was suggested that the mass lesion in myometrium may be secondary to residual placenta. A discussion was made with the patient and hysterectomy was planned. Explorative operation showed that urinary bladder was adhered to anterior uterine wall at the lower uterine segment and there was a formation consistent with placenta percreta extending beyond the serosa and invading the urinary bladder at the site of previous caesarean section. Placenta was detached from urinary bladder and hysterectomy was performed. Urinary bladder was repaired. The hysterectomy material was sent for pathological examination, which revealed a lesion consistent with placenta localized to myometrium and extending to serosa at the level of isthmus, and a hematoma opening to endometrial cavity medial to this lesion (Fig 3). Histopathological examination revealed that chorionic villi invaded myometrium and extended to serosa (Fig 4), thus confirming the diagnosis of placenta percreta.

## Discussion

Placenta percreta is a disorder that results from regional or insufficient diffusion of decidua basalis and is characterized by placenta passing beyond myometrium to reach serosa. Approximately, 5% of the cases with abnormal placentation consist of placenta percreta. Nearly all cases of placenta percreta are diagnosed in 3rd trimester. Its incidence has been on the rise with each passing day as a result of the increase in the number of caesarean section operations (3).

Risk factors for placenta percreta include previous surgery (caesarean section, myomectomy or curettage), abnormal placental localizations, advanced maternal age, grand multiparity, Asherman’s syndrome, endometritis, adenomyosis, endometriosis, and submucous leiomyoma. Our patient had 2 of these risk factors, including advanced age and previous caesarean sections. Early use of USG and MRI to establish an early and accurate diagnosis, and to determine the appropriate treatment modality are of paramount importance in reducing the morbidity (3, 5, 6). Ultrasonographic findings that may aid in diagnosis include loss of normal hypoechoic zone of the retroplacental myometrium, thinning and interruption of the hyperechogenicity between uterine serosa and urinary bladder, and presence of a focal exophytic mass suggesting neighboring organ invasion, especially of the urinary bladder. MRI is frequently used in combination with USG. The sensitivity and specificity reported for MRI in detecting abnormal placentation are 80-88% and 65-100%, respectively (7). However, the ability of MRI to diagnose placental invasion of myometrium is still dependent on the experience of the interpreter. Sometimes, even combined use of USG and MRI may fail to diagnose abnormal placentation.

Two strategies have been proposed for treatment of placenta percreta, namely hysterectomy and conservative treatment. Although, hysterectomy is the first line treatment modality, it may prove insufficient in achieving hemostasis in cases with advanced and severe invasion of adjacent structures. Thus, hemodynamically stable patients with placenta percreta may be conservatively treated with methotrexate (8). Uterine artery embolization is another conservative method that may be used for patients that wish to preserve their fertility. As our patient had no desire to preserve her fertility, we proceeded with hysterectomy after discussion about various treatment modalities. The anterior wall of urinary bladder to which placenta was adhered, was repaired. No complication developed during the operation.

Our literature search yielded very few cases with placenta percreta diagnosed in the first and second trimester of pregnancy. Massive bleeding may develop following curettage performed after incomplete abortion and hysterectomy may be required to stop the bleeding (9, 10). Gupta et al. reported that all patients with placenta accreta underwent hysterectomy, after curettage was performed for incomplete abortion in such patients (10). While iatrogenic uterine rupture may develop due to curettage performed for the treatment of incomplete abortion, spontaneous rupture may ensue as pregnancy progresses (11, 12) in the patients with placenta percreta.

In our case, incomplete abortion at the 6^th^ week of pregnancy, leading to an early curettage, may have prevented progressive placental growth and further complications. No massive bleeding was observed during curettage and an urgent hysterectomy was not needed. Furthermore, an increased serum β-HCG level in the preoperative period, history of curettage for incomplete abortion 2 months back, no history of sexual intercourse after curettage, history of 2 previous cesarean deliveries and demonstration of placental residue in the uterine cavity on USG and MRI all led to the correct diagnosis. To our knowledge, there is no case diagnosed with placenta percreta as early as 6 weeks of gestation, in English literature.

In conclusion placenta percreta is one of the most important complications of pregnancy with serious morbidity and mortality. First trimester diagnosis is quite difficult. USG and MRI are diagnostic adjuncts. Unexplained protracted vaginal bleeding after curettage for incomplete abortion should raise the suspicion of placenta percreta.

More importantly, uterine rupture may take place in these patients during curettage, leading to shock secondary to abundant hemorrhage. For this reason, it is recommended to perform the curettage in a fully equipped healthcare facility where blood transfusion and hysterectomy can be carried out.
